# First establishment of a new table-rotated-type hybrid emergency room system

**DOI:** 10.1186/s13049-018-0532-z

**Published:** 2018-09-17

**Authors:** Hiroaki Watanabe, Yoshihide Shimojo, Eiji Hira, Shunsuke Kuramoto, Tomohiro Muronoi, Kazuyuki Oka, Akihiko Kidani

**Affiliations:** Department of Acute Care Surgery, Shimane University Faculty of Medicine, 89-1 Enya-cho, Izumo, Shimane 693-8501 Japan

**Keywords:** Hybrid ER, Table-rotated-type hybrid ER system, IVR-CT-operation system, Trauma resuscitation, Whole-body CT

## Abstract

The hybrid emergency room (hybrid ER) system was first established in 2011 in Japan. It is defined as an integrated system including an ER, emergency computed tomography (CT) and interventional radiology (IVR) rooms, and operating rooms. Severe trauma patients can undergo emergency CT examinations and therapies (surgeries) without being transferred. The hybrid ER system is attracting attention because trauma resuscitation using this system has been reported to potentially improve the mortality rate in severe trauma patients. In August 2017, we established a new table-rotated-type hybrid ER to facilitate surgical functions. Herein, we introduce a new table-rotated-type hybrid ER consisting of an IVR–CT–operating room system and discuss its efficiency and feasibility for trauma resuscitation, including surgery and IVR. This system includes four new concepts: (1) to secure a wide working space during trauma resuscitation by reconsidering the arrangement of the C-arm, (2) ensure an air-conditioned operating room in the hybrid ER, (3) adopt an operating table but not interventional radiology table, and (4) prepare a trauma bay with three additional beds for multiple victims. This hybrid ER system also adopted the rotated-type table to secure a wide working space during the resuscitation phase. The C-arm was located away from the patients and placed on the wall opposite to the CT gantry, in contrast to that in previous systems. If patients needed an emergency IVR, the table was just rotated, and the IVR could be conducted immediately. This improvement can secure a wide working space in the hybrid ER. Moreover, the patient table was also a surgical operating table, and the hybrid ER system had an air-conditioned operating room (class 10,000). In the anticipation of many trauma patients being transported to the ER, a new trauma bay with three additional beds next to the hybrid ER was established, which also had an air-conditioned operating room. This new rotated-type hybrid ER system facilitates efficient surgical functions during trauma resuscitation and can secure a wide working space for the medical team to immediately perform resuscitative procedures and IVRs without delay.

## Background

Since whole-body computed tomography (CT) imaging during trauma resuscitation was reported to significantly increase the probability of survival in trauma patients, it has been recommended in early trauma care in emergency departments (ERs) [1]. On the basis of this theory, the hybrid emergency room (hybrid ER) was first established in 2011 in Japan [2], and trauma care in a hybrid ER has been shown to potentially be associated with improved survival in severe trauma patients [3]. Moreover, Kinoshita et al. reported that immediate diagnosis by CT imaging and rapid massive bleeding control without transferring the patients because of the installed hybrid ER system might significantly improve mortality in severe trauma patients [4]. At present, 10 trauma centers in Japan have a hybrid ER system, because using hybrid ER during trauma care is beneficial for severe trauma patients.

In general, a hybrid ER is defined as an integrated system that includes the ER, emergency CT room, interventional radiology (IVR) room, and operating room. The most important feature of this rotated-type hybrid ER system is that it allows severe trauma patients to undergo emergency CT examinations and therapies such as surgeries immediately, without transfer to other rooms. In the previous trauma workflow, trauma patients were assessed by emergency physicians in the ER; then, if necessary, they were transferred to the CT room; and finally, they were transferred to the operating room or IVR room if they needed operations or IVR. Until definitive therapy, patients had to be transferred to several rooms repeatedly. However, if a hybrid ER is used, massive bleeding can be controlled immediately after CT examination via initial trauma survey without transferring the patients because this ER functions as an operating room, an IVR room, and a CT room.

Several existing hybrid ER systems have a CT gantry and C-arm beside the treatment table. IVR is not frequently conducted during an initial trauma survey. Many trauma physicians and surgeons would like to secure a wide working space in the ER. Therefore, we established a new table-rotated-type hybrid ER to secure a wide working space and enhance immediate surgical functions during trauma resuscitation. Herein, we introduce this new type of hybrid ER system for trauma resuscitation.

## Main text

Our trauma center was established in April 2016 in Shimane University Hospital. Approximately 2200 trauma patients were transferred to or visited our trauma center every year. In 2017, 465 patients, including 279 who had severe trauma (AIS of ≥3), were admitted to our hospital. In August 2017, we constructed a new trauma center building including one hybrid ER, three-bed trauma bays, and two operating rooms.

**Fig. 1:**
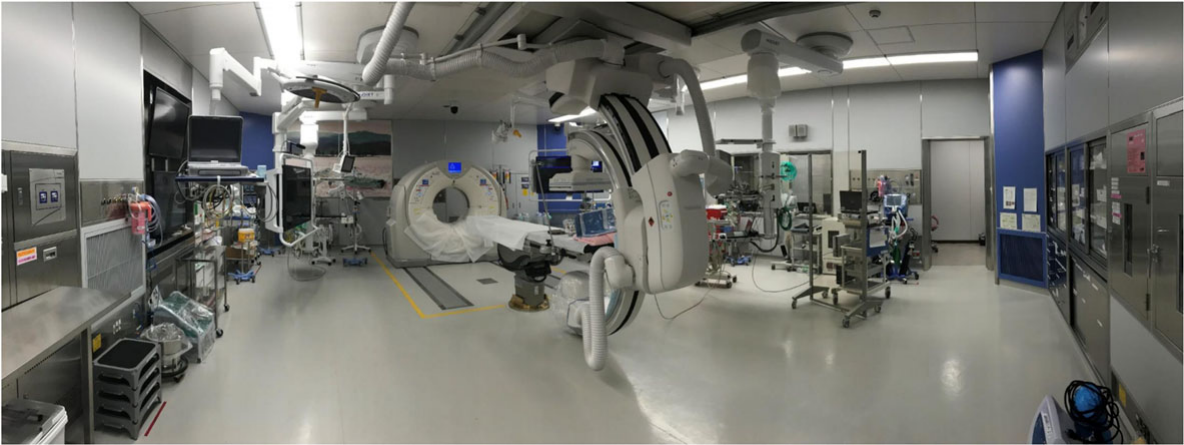
A photograph of rotated type hybrid ER system (Canon Medical Systems Corp., Ohtawara, Japan). C-arm is located on the wall at the opposite side of the CT gantry; therefore, this system can secure wide working space in resuscitation phase

The hybrid ER system was located on the first floor (Fig. 1). Important concepts of our hybrid ER system are (1) to secure a wide working space for resuscitation, (2) to ensure that the operating room is air conditioned in the hybrid ER, (3) to adopt an operating table but not intervention table, and (4) to prepare the trauma bay with three additional beds for multiple victims.

**Fig. 2:**
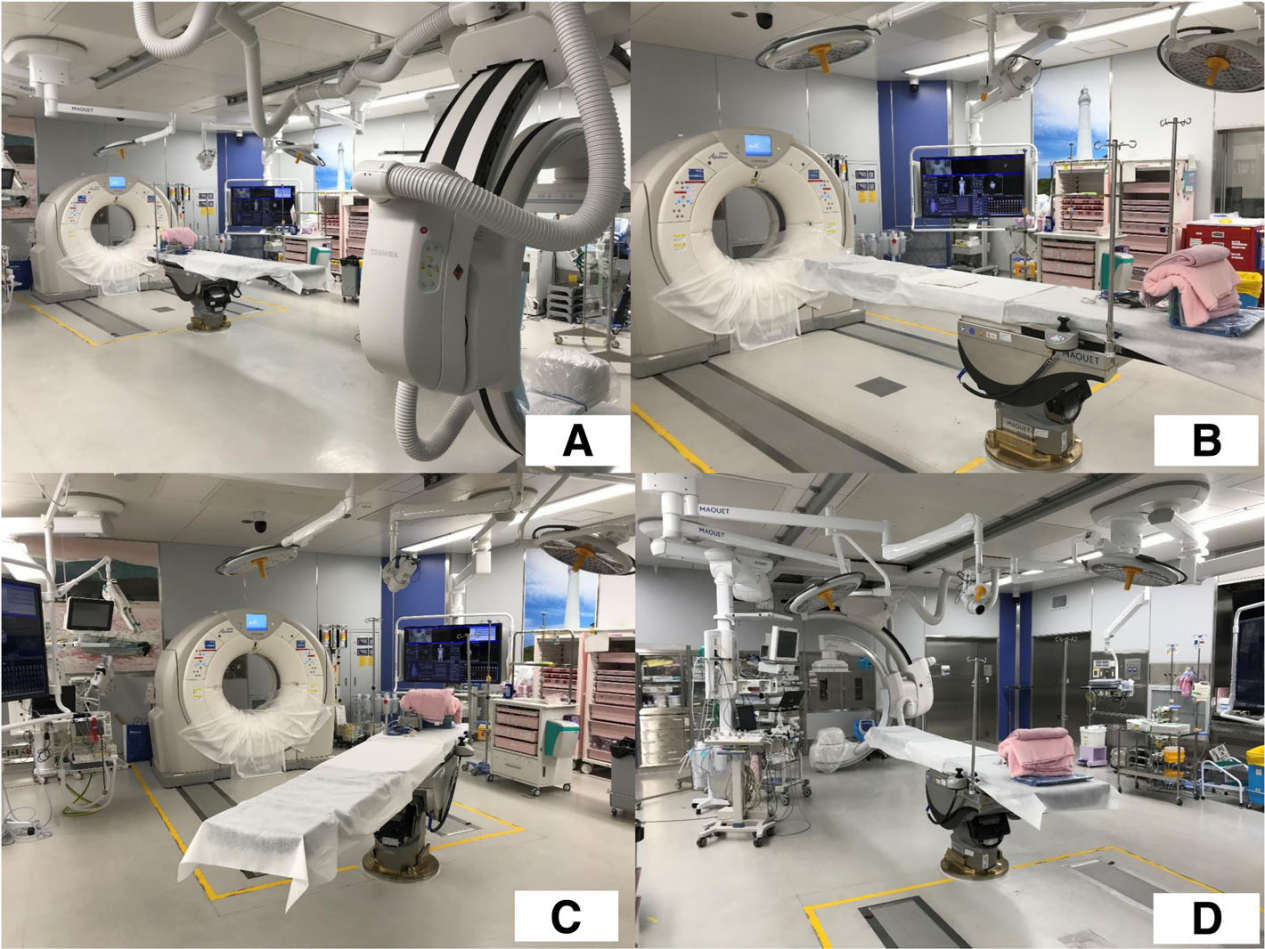
**a** shows that C-arm and CT gantry are located at the opposite side. **b** shows CT mode in which primary survey and CT are performed. **c** shows rotating patient table. **d** shows intervention mode in which surgeries and interventional radiology are performed after rotating the patient table

Our hybrid ER system was of the table rotated type, which is a new concept, to secure a wide working space during the resuscitation phase. In the previous existing hybrid ER system, the C-arm was located beside the patients. However, C-arms are not frequently used. Therefore, they were placed on the wall opposite the CT gantry in our system (Fig. 2a), and if patients needed an IVR, the table could be rotated (Fig. 2c). Thus, emergency physicians or trauma surgeons could perform the initial trauma care in a wide space.

Basically, severe trauma patients are directly transported to this hybrid ER system by the emergency medical services. Patients are transferred to the operating table so that their heads are located on the side with the CT system. This position is called “CT mode position” (Fig. 2b). In the CT mode position, primary survey is performed, and physiological damage including massive bleeding is detected by CT scanning. If massive bleeding such as massive hemothorax, abdominal bleeding, and retroperitoneal bleeding are detected on CT scanning, the patient table is rotated for the intervention and emergency procedures, which are immediately performed without transferring the patients. This position is called “intervention mode.” Emergency operation and IVR are basically performed in the intervention mode (Fig. 2d). If the patient has severe brain injury and massive abdominal bleeding, both injuries can be immediately detected by CT scanning and both surgeries can be immediately performed simultaneously.

Cleaned instruments for the operation and IVR set are frequently deployed together during trauma resuscitation. This rotated type system is excellent for securing wide working space with careful consideration to various situations.

Our hybrid ER system has an air-conditioned operating room (class 10,000), but many hybrid ER systems are not air conditioned. Moreover, the patient table in our hybrid ER system consists of an operating table, whereas that in other hybrid ER systems consist of interventional radiology table. Our hybrid ER is installed in the emergency department, but it is a complete operating room. Therefore, all types of operations including craniotomies can be performed.

**Fig. 3:**
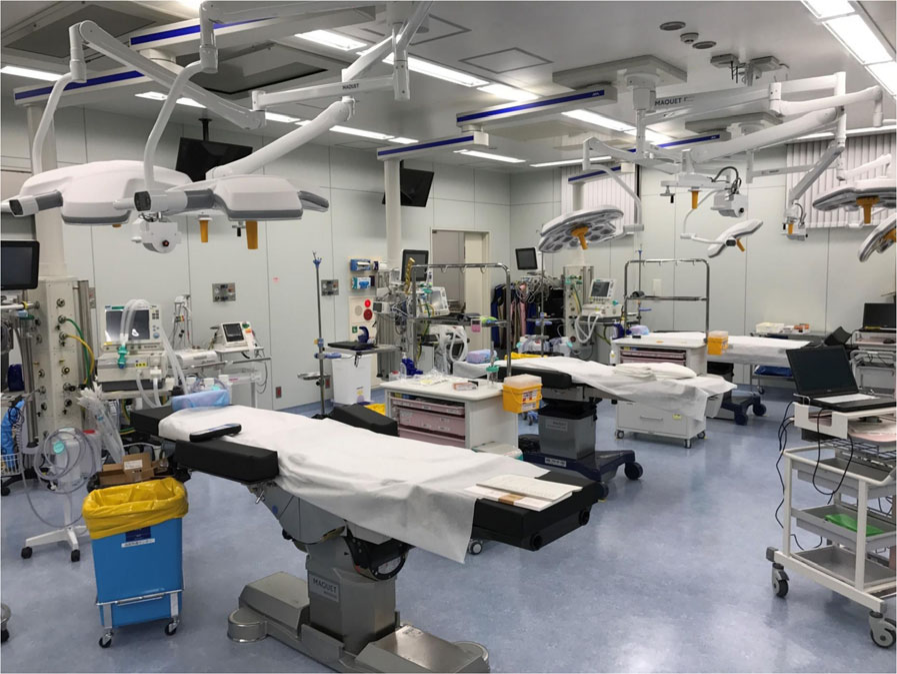
A photograph showing a trauma bay including three additional beds next to the hybrid ER. An air-conditioned operating room is also found in this area. (class 10,000)

The hybrid ER system is an excellent system for the immediate intervention of severe trauma patients with massive bleeding because immediate detection of the bleeding points and hemostasis can be performed without the need for transfer of the patients. However, this system cannot treat two patients with severe traumas at the same time. Several patients are often simultaneously transported during a traffic accident. Therefore, we installed a trauma bay with three beds next to the hybrid ER (Fig. 3). In this way, we can cater to four severe trauma patients at the same time. The trauma bay room also has an air-conditioned operating room (class 10,000), and an emergency operation can also be performed here.

To operate this system, we need several medical personnel, including a trauma surgeon, a trauma nurse, a radiologist, and an interventionist, among others. Therefore, a command and control system should be established. The commander must appropriately coordinate with the trauma team.

## Conclusions

We established a new hybrid ER system with a rotated-type table and an IVR–CT–operating system and reported the efficiency and feasibility of this new system. This new rotated-type hybrid ER system facilitates efficient surgical functions during trauma resuscitation and can secure a wide working space when performing operations, IVR, and resuscitations immediately without delay.

## Abbreviations

CT: computed tomography
Hybrid ER: hybrid emergency room
IVR: interventional radiology
